# Anti-OSM Antibody Inhibits Tubulointerstitial Lesion in a Murine Model of Lupus Nephritis

**DOI:** 10.1155/2017/3038514

**Published:** 2017-05-24

**Authors:** Qingjuan Liu, Yunxia Du, Kejun Li, Wei Zhang, Xiaojuan Feng, Jun Hao, Hongbo Li, Shuxia Liu

**Affiliations:** ^1^Department of Pathology, Key Laboratory of Kidney Diseases of Hebei Province, Hebei Medical University, Shijiazhuang, Hebei 050017, China; ^2^Department of Ophthalmology, People's Hospital of Hebei Province, Shijiazhuang, Hebei 050000, China

## Abstract

The purpose of this study was to investigate the role of oncostatin M (OSM) in tubulointerstitial lesion (TIL) in lupus nephritis (LN). We found that OSM was highly expressed in the renal tissue of LN mice. OSM is one of the interleukin-6 cytokine family members. In order to clarify the role and mechanism of OSM in LN, mice with LN were treated with anti-OSM antibody or isotype antibody. We evaluated the tubular epithelial-mesenchymal transdifferentiation (EMT) by detecting the E-cadherin, *α*-smooth muscle actin (*α*-SMA), and fibronectin (FN) expression. We analyzed the inflammation by observing the monocyte chemotactic factor-1 (MCP-1) and intercellular adhesion molecule (ICAM-1) expression and calculated the tubulointerstitial fibrosis area by Masson staining. The results showed that anti-OSM antibody, rather than isotype antibody, improved EMT, inflammation, and tubulointerstitial fibrosis. In addition, the signal transducer and activator of transcription (STAT) 1 and STAT3 signaling was activated by tyrosine phosphorylation in LN mouse renal tissue, indicating that the phosphorylated STAT1 (p-STAT1) and p-STAT3 were involved in kidney injury. Moreover, decreased p-STAT3 instead of p-STAT1 has been observed after anti-OSM antibody injection. Thus, we concluded that OSM is associated with TIL in lupus nephritis, which may be connected with the activation of STAT3 rather than that of STAT1.

## 1. Introduction

Systemic lupus erythematosus (SLE) is a systemic disease that can affect almost all organs in the body. During the course of the disease, there is a high incidence of kidney damage, known as lupus nephritis (LN). Kidney damage is a significant cause of death and disability in SLE patients [1, 2] and includes glomerular, tubular, and renal interstitium and vessel injuries. Among them, the incidence of tubulointerstitial lesion (TIL) is as high as 50% to 67% [3]. TIL manifests as tubular epithelial cell degeneration, inflammation, and tubulointerstitial fibrosis. It was reported that TIL may also be an important independent risk factor for LN and may affect the prognosis of it [4]. Therefore, it is vital to explore the mechanism of TIL and identify ways to inhibit its progression in LN.

Cytokines are necessary for the occurrence and progression of LN. Oncostatin M (OSM) is a pleiotropic cytokine that belongs to the interleukin-6 cytokine family and is produced from activated T cells and monocytic cells [5]. It has been reported that OSM is positively correlated with SLE activity and may be a useful marker for it [6]. Importantly, mounting evidence shows that OSM is also involved in pathological conditions in kidney diseases. For instance, studies by Nightingale et al. and Pollack et al. [7, 8] showed that human proximal tubular epithelial cells undergo epithelial-mesenchymal transdifferentiation (EMT) in response to OSM. Moreover, treatment with anti-OSM antibody ameliorated renal fibrosis in obstructive nephropathy [9]. These studies strongly suggest that OSM has profibrotic properties. However, Sarközi et al. proposed that OSM could inhibit transforming growth factor- (TGF-) *β*1-induced extracellular matrix production in proximal tubule cells and could, thus, have a dual role in the regulation of cellular mechanisms associated with renal tubulointerstitial fibrogenesis [10, 11]. Further research is needed to study the role of OSM in kidney diseases.

In the present study, we aimed to examine the expression of OSM in the renal tissue of LN mice and to evaluate the possible role and mechanism of OSM in TIL.

## 2. Materials and Methods

### 2.1. Animals and Treatment

MRL/MpJ and MRL/MpJ-Fas/lpr (MRL/lpr) mice were used in this study. An MRL/lpr mouse model was firstly established by Murphy and Roths by using multiple inbred mice. This model bears the lymphocyte proliferation gene (lpr) mutation, the result of a retroviral insertion in Fas gene, leading to a systemic autoimmune disease similar to human SLE, including antibody production, immunocomplex formation, and nephritis. The renal pathological changes in MRL/lpr mice are very similar with human lupus nephritis. Therefore, this kind of model is an ideal animal model to be used in the investigation of SLE. MRL/MpJ mice, as the control of MRL/lpr mice, have the same manifestation of autoimmune abnormalities. But the emergence of such symptoms is much later than that in MRL/lpr mice.

Female MRL/MpJ mice were designated as the control group. And female MRL/lpr mice were randomly divided into the LN group, the anti-OSM antibody group, and the isotype antibody group. There were six mice (weight, 45–55 g) in each group. The mice were provided by the Model Animal Research Center of Nanjing University. All experiments with animals were approved by the Animal Care and Use Committee of Hebei Medical University. An intraperitoneal injection of anti-OSM polyclonal antibody (R&D Systems, Minneapolis, MN, USA) or isotype antibody (normal IgG) (R&D Systems) was administered weekly from 20 to 24 weeks of age at a dose of 1 mg/kg. All of the animals were housed in an environment with fixed light, temperature, and humidity as well as freely accessible food and water. The mice were euthanized at 24 weeks. Serum, urine, and renal cortex samples were collected for further study.

### 2.2. Histopathological Examination

After deparaffinization of the paraffin sections, Masson staining was performed to evaluate tubulointerstitial fibrosis. Collagen fibers were stained blue. The area of collagen deposition was determined as the percentage of the blue-stained area to the whole area.

### 2.3. Quantitative Real-Time PCR (qPCR)

Total RNA was isolated from the renal cortex using TRIzol reagent. Complementary DNA was synthetized from 1 *μ*g total RNA using a PrimeScript™ RT reagent Kit (Takara, Mie, Japan) following the manufacturer's recommendations. qPCR was performed with SYBR Remix Ex Taq™ II (Takara). The qPCR thermal cycling condition for all reactions was 95°C for 30 s, followed by 40 cycles of 95°C for 5 s and 55°C for 30 s. All target gene primers were purchased from Sangon Biotech (Shanghai, China). The results were expressed as relative expression levels to GAPDH gene. The CT values were used as the original data to calculate the relative expression levels of different genes by the 2^−ΔΔCT^ method.

### 2.4. Enzyme-Linked Immunosorbent Assay

The homogenate of the renal cortex was centrifuged at 1000 ×g for 10 min at 4°C, and the supernatants were collected to detect the OSM expression. The level of OSM was quantified using a competitive sandwich ELISA kit (Abgent, San Diego, CA, USA) according to the manufacturer's directions.

### 2.5. Immunohistochemistry

The kidneys were dissected and fixed immediately in fixative for 24 h. The kidneys were then dehydrated, processed, and embedded in paraffin. For immunohistochemistry, 4 *μ*m sections were deparaffinized and rehydrated. Antigen recovery was performed by a microwave. Next, the sections were incubated with primary antibodies against E-cadherin and *α*-smooth muscle actin (*α*-SMA), respectively, overnight at 4°C. The antibodies were purchased from Proteintech Group (Chicago, IL, USA). On the following day, after incubating sequentially with a biotinylated secondary antibody and horseradish peroxidase-conjugated streptavidin for 30 min at 37°C, the sections were stained with 3,3-diaminobenzidine (DAB). The negative controls were prepared by replacing the primary antibody with phosphate-buffered saline.

### 2.6. Western Blot Analysis

Tissue lysates were prepared as in previous studies [12]. The protein extracts from the renal cortex were separated by 10% sodium dodecyl sulfate-polyacrylamide gel electrophoresis and then transferred to polyvinylidene fluoride membranes. The membranes were blocked with 5% nonfat milk for 2 h at 37°C and then were incubated overnight at 4°C with anti-E-cadherin, *α*-SMA, monocyte chemotactic factor-1 (MCP-1), intercellular adhesion molecule (ICAM-1), phosphorylated signal transducer, and activator of transcription 1 (p-STAT1), p-STAT3, total STAT1, total STAT3, or *β*-actin antibodies. The anti-*β*-actin antibody was purchased from ZSGB-BIO (Beijing, China). The anti-p-STAT and total STAT antibodies were purchased from Cell Signaling (Boston, MA, USA). The other antibodies were purchased from Proteintech Group (Chicago, IL, USA). Thereafter, the membranes were incubated with a horseradish peroxidase-conjugated goat anti-rabbit IgG antibody (ZSGB-BIO) and imaged using the Odyssey Infrared Imaging System (LI-COR, Lincoln, NE, USA).

### 2.7. Statistical Analysis

The quantitative data are expressed as the mean ± standard deviation and analyzed by one-way analysis of variance (ANOVA) with the Student-Newman-Keuls test. Statistical significance was set at *P* < 0.05.

## 3. Results

### 3.1. Level of OSM in the Kidney

**Figure 1:**
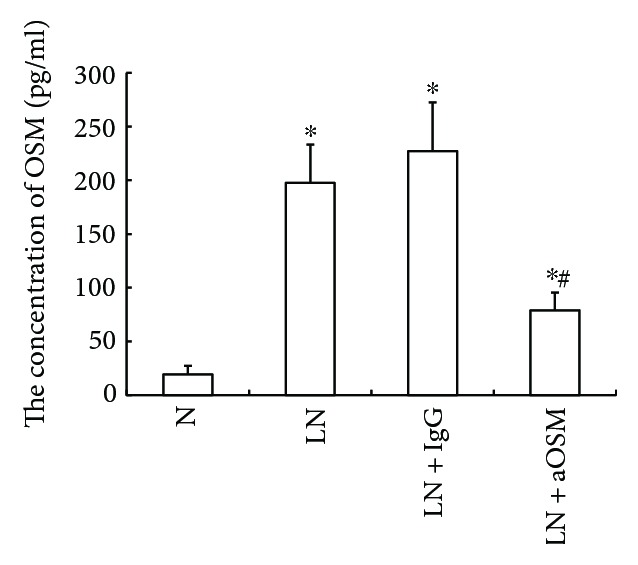
The expression level of OSM in renal tissue by ELISA. Compared with that in normal control mice, the expression level of OSM increased in LN mice. Anti-OSM antibody instead of normal IgG reduced the OSM level. N = normal control mice; LN = lupus nephropathy mice; LN + IgG = LN mice treated with normal IgG; LN + aOSM = LN mice treated with anti-OSM antibody. The values are expressed as the means ± SD. ^∗^*P* < 0.05 versus normal control mice (N), ^#^*P* < 0.05 versus lupus nephropathy mice (LN).

Taking into account the dual role of OSM in renal fibrosis, we first detected the level of OSM in the renal cortex by ELISA (Figure 1). The data suggested that OSM expression level was markedly elevated by about 10.23-fold in the kidneys of MRL/lpr mice compared with those of MRL/MpJ mice (*P* < 0.05). However, MRL/lpr mice, treated with anti-OSM antibody rather than isotype antibody, had approximately 60% decrease in the level of OSM as compared to MRL/lpr mice (*P* < 0.05).

### 3.2. Effects of Anti-OSM Antibody on Renal Function

**Figure 2:**
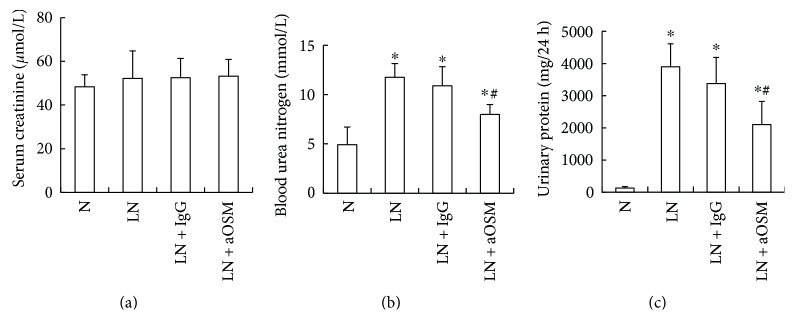
The effects of anti-OSM antibody on the levels of serum creatinine, 24 h urinary protein, and blood urea nitrogen. (a) The level of serum creatinine. (b) The level of blood urea nitrogen. (c) The level of 24 h urinary protein. N = normal control mice; LN = lupus nephropathy mice; LN + IgG = LN mice treated with normal IgG; LN + aOSM = LN mice treated with anti-OSM antibody. The values are expressed as the means ± SD. ^∗^*P* < 0.05 versus normal control mice (N); ^#^*P* < 0.05 versus lupus nephropathy mice (LN).

Serum creatinine (SCr), blood urea nitrogen (BUN), and 24-hour urinary protein (Upro) were detected (Figure 2). There was no difference in the level of SCr between the control and LN groups (*P* > 0.05), but the amount of BUN and Upro in MRL/lpr mice was significantly increased (BUN: 2.39-fold higher than those in MRL/MpJ mice, Upro: 30.87-fold higher than those in MRL/MpJ mice; *P* < 0.05). And treatment with anti-OSM antibody decreased the amount of BUN and Upro (BUN: 32.06% decrease; Upro: 46.04% decrease). However, there was no change in the level of BUN and Upro after isotype antibody injection (*P* > 0.05).

### 3.3. Effects of Anti-OSM Antibody on Phosphorylation and Activation of STAT1 and STAT3

**Figure 3:**
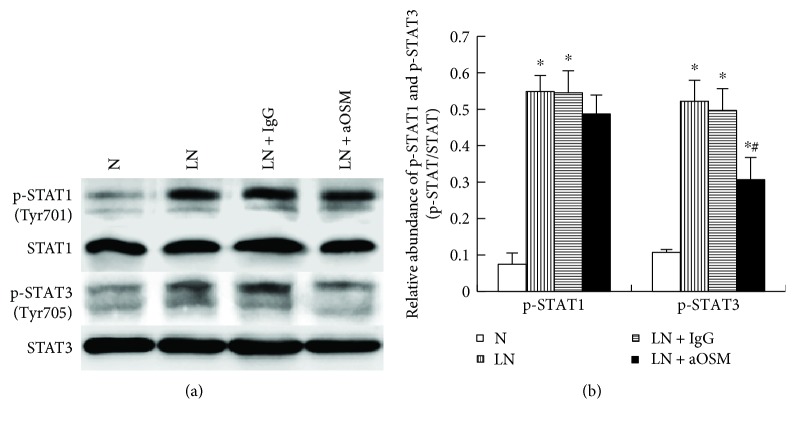
The activation and expression of STAT1 and STAT3 in renal tissue. (a) The expression of STAT1 and p-STAT1 was analyzed by Western blot assay and quantified by densitometry (mean ± SD). (b) The expression of STAT3 and p-STAT3 was analyzed by Western blot assay and quantified by densitometry (mean ± SD). N = normal control mice; LN = lupus nephropathy mice; LN + IgG = LN mice treated with normal IgG; LN + aOSM = LN mice treated with anti-OSM antibody. ^∗^*P* < 0.05 versus normal control mice (N); ^#^*P* < 0.05 versus lupus nephropathy mice (LN).

STAT signaling is one of the main signals activated by OSM. In order to clarify whether STAT1 and STAT3 are involved in OSM-mediated kidney injury, we examined the effect of anti-OSM antibody on the activation and expression of STAT1 and STAT3 in the kidneys of MRL/lpr mice. As shown in Figure 3, the levels of p-STAT1 (0.07 ± 0.03) and p-STAT3 (0.11 ± 0.01) were very low in the control group. But STAT1 and STAT3 were markedly activated by tyrosine phosphorylation (p-STAT1: 0.55 ± 0.04; p-STAT3: 0.52 ± 0.06), and total STAT expression did not change significantly in LN mice (*P* > 0.05). The administration of anti-OSM antibody suppressed the expression level of p-STAT3 by 41.21% (*P* < 0.05) but only induced a small, not statistically significant decrease in the expression of p-STAT1 (*P* > 0.05). However, total STAT1 and STAT3 expression were not affected by this treatment (*P* > 0.05).

### 3.4. Anti-OSM Antibody Increased the Expression of E-Cadherin but Decreased the Expression of *α*-SMA in LN Mice

**Figure 4:**
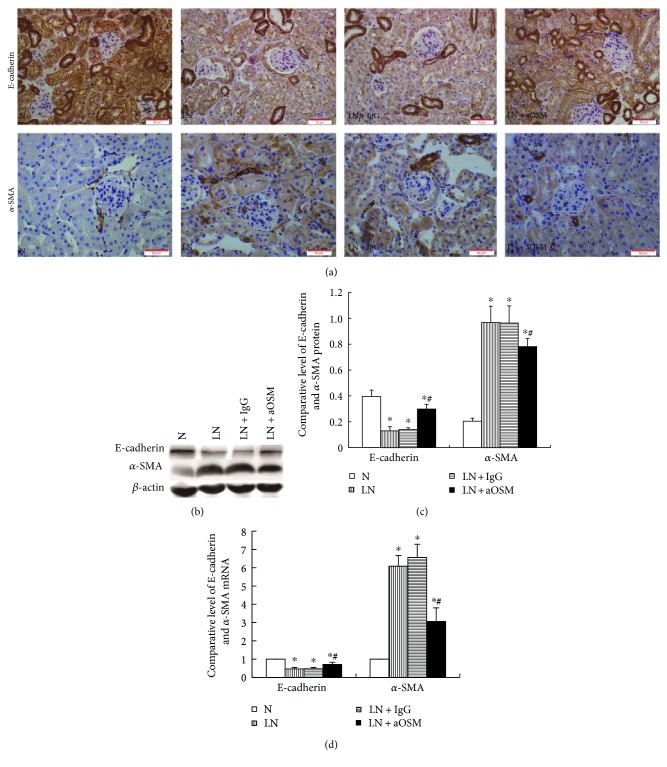
Protein and mRNA expression of E-cadherin and *α*-SMA. (a) Immunohistochemistry for E-cadherin and *α*-SMA. (b, c) The expression of E-cadherin and *α*-SMA was analyzed by Western blot assay and quantified by densitometry (mean ± SD). (d) the expression of E-cadherin and *α*-SMA mRNA was analyzed by qPCR (mean ± SD). N = normal control mice; LN = lupus nephropathy mice; LN + IgG = LN mice treated with normal IgG; LN + aOSM = LN mice treated with anti-OSM antibody. ^∗^*P* < 0.05 versus normal control mice (N); ^#^*P* < 0.05 versus lupus nephropathy mice (LN).

Tubular epithelial-myofibroblast transdifferentiation (EMT) is an important event in tubulointerstitial fibrosis. EMT is characterized by the downregulation of epithelial marker proteins such as E-cadherin, cytokeratin, and zonula occludens 1 and upregulation of mesenchymal markers including vimentin, *α*-SMA, and fibroblast-specific protein 1. In this study. we examined the expression of E-cadherin and *α*-SMA in the renal cortex. The results by immunohistochemistry staining (Figure 4(a)) showed that E-cadherin was located in the plasmalemma and cytoplasm of tubular epithelial cells. The proximal tubule rather than the distal tubule showed expression change of E-cadherin. In normal control mice, *α*-SMA was expressed in the vascular wall only, whereas it was expressed both in the vascular wall and the proximal tubule in LN mice. MRL/lpr mice had approximately 67.10% decrease in the level of E-cadherin expression and 372.50% increase in the level of *α*-SMA expression compared with MRL/MpJ mice. In contrast, anti-OSM antibody treatment resulted in an elevated expression of E-cadherin (2.29-fold increase compared with that in the LN group, *P* < 0.05) and a depressed expression of *α*-SMA (19.25% decrease compared with that in the LN group, *P* < 0.05) (Figures 4(b) and 4(c)). The expression of E-cadherin and *α*-SMA at mRNA level examined by qPCR showed similar results as their protein (Figure 4(d)).

### 3.5. Anti-OSM Antibody Suppressed the Expression of MCP-1 and ICAM-1

**Figure 5:**
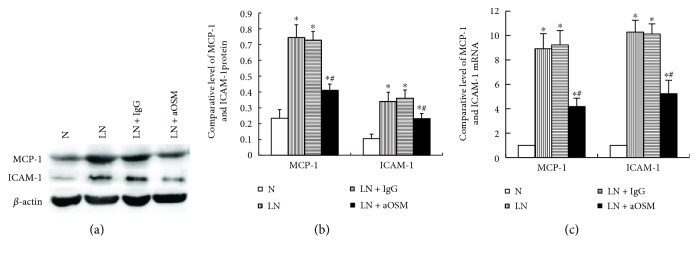
Protein and mRNA expression of MCP-1 and ICAM-1. (a, b) The expression of MCP-1 and ICAM-1 was analyzed by Western blot assay and quantified by densitometry (mean ± SD). (c) The expression of MCP-1 and ICAM-1 mRNA was analyzed by qPCR (mean ± SD). N = normal control mice; LN = lupus nephropathy mice; LN + IgG = LN mice treated with normal IgG; LN + aOSM = LN mice treated with anti-OSM antibody. ^∗^*P* < 0.05 versus normal control mice (N); ^#^*P* < 0.05 versus lupus nephropathy mice (LN).

The increased infiltration of monocytes/macrophages in renal tissue is the most common pathology of kidney diseases. The mechanism of monocyte/macrophage infiltration is not clear, but the upregulation of chemokine and adhesion molecule expression is considered to be an important factor leading to the monocyte/macrophage infiltration in renal tissue. We examined the expression of MCP-1 and ICAM-1 in each group of mouse renal tissues. As indicated in Figures 5(a) and 5(b), basal levels of MCP-1 (0.23 ± 0.05) and ICAM-1 (0.10 ± 0.03) were detected in the renal tissue of MRL/MpJ mice. The kidney injury of MRL/lpr mice resulted in a marked increase in their expression (MCP-1: 3.18-fold higher expression than those in MRL/MpJ mice, ICAM-1: 3.25-fold higher expression than those in MRL/MpJ mice; *P* < 0.05). Anti-OSM antibody treatment inhibited the protein expression of MCP-1 by 44.91% and ICAM-1 by 32.03% (*P* < 0.05). Moreover, we observed the effect of anti-OSM antibody on the expression of MCP-1 and ICAM-1 mRNA and found that in MRL/lpr mice, the mRNA level of MCP-1 and ICAM-1 also increased (MCP-1 mRNA: 8.92-fold higher than those in MRL/MpJ mice, ICAM-1 mRNA: 10.28-fold higher than those in MRL/MpJ mice; *P* < 0.05). However, anti-OSM antibody administration ameliorated this response (*P* < 0.05) (Figure 5(c)).

### 3.6. Anti-OSM Antibody Attenuated Deposition of ECM Components

**Figure 6:**
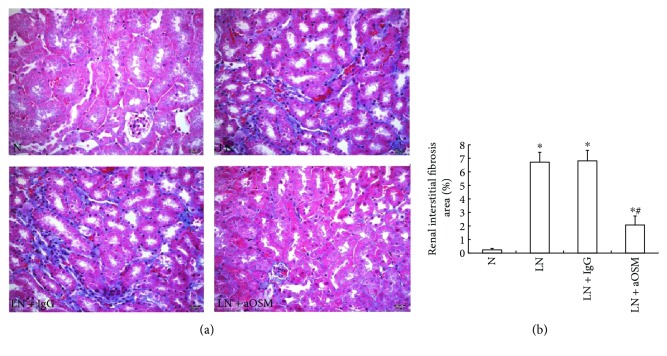
Pathological changes in renal interstitial fibrosis observed by Masson staining. (a) Anti-OSM antibody treatment reduced the blue area. (b) The data are expressed as the mean ± SD. N = normal control mice; LN = lupus nephropathy mice; LN + IgG = LN mice treated with normal IgG; LN + aOSM = LN mice treated with anti-OSM antibody. ^∗^*P* < 0.05 versus normal control mice (N); ^#^*P* < 0.05 versus lupus nephropathy mice (LN).

The deposition of extracellular matrix is the major feature of renal fibrosis. In order to identify whether OSM participates in tubulointerstitial fibrosis, we evaluated the effect of anti-OSM antibody on the interstitial collagen fibrils by Masson staining. A marked increase (28.50-fold higher than those in MRL/MpJ mice, *P* < 0.05) in aniline blue-positive areas (blue area) was observed in the renal interstitium in MRL/lpr mice, and the administration of anti-OSM antibody reduced such areas by 69.08% (*P* < 0.05) (Figure 6).

## 4. Discussion

Using anti-OSM antibody, we provided evidence that OSM participates in the TIL of lupus nephropathy. We have shown that anti-OSM antibody increased the expression of E-cadherin and decreased the expression of *α*-SMA, suppressed the expression of MCP-1 and ICAM-1, attenuated renal fibrosis, and partially improved 24-hour urinary protein excretion and BUN production. In addition, the effects of OSM in the TIL of LN mice may be connected with the activation of STAT3 instead of STAT1 signaling.

We previously found that OSM induced EMT and extracellular matrix secretion of tubular epithelial cells in vitro via the activation of the Janus kinase (JAK)/STAT pathway [13]. It suggested that depending on the activation of STAT, OSM is involved in the tubulointerstitial damage. This is consistent with the previous findings of Nightingale et al. and Pollack et al. [7, 8]. In this study, we examined the role of OSM in TIL in LN mice. The results demonstrated that OSM participates in TIL in LN mice. Anti-OSM antibody treatment could ameliorate TIL by improving EMT, inflammation, and fibrosis, suggesting that OSM could also be related to the progression of TIL in vivo.

We then explored the mechanism of OSM in TILs. The JAK/STAT signaling pathway is one of the pivotal signals activated by OSM. The STAT protein family comprises six members, STAT1 through STAT6. Both STAT1 and STAT3 can be activated by OSM [7]. Earlier reports indicated that the activation of STAT1 and STAT3 often demonstrates different roles. STAT3 has been shown to promote tumor progression, whereas STAT1 tends to suppress it [14, 15]. In order to confirm the role of STAT signaling in TILs and the relationship between STAT and OSM, we detected the expression of p-STAT1 and p-STAT3 in renal tissue. The results showed that both STAT1 and STAT3 are phosphorylated and activated in LN mice. Nevertheless, anti-OSM antibody inhibited mainly the phosphorylation and activation of STAT3 instead of STAT1. This finding indicated that the therapeutic effect of anti-OSM antibody in TIL attributed to the activation of STAT3 rather than that of STAT1.

However, Sarkozi found that besides the OSM-mediated induction of morphological alterations indicative of renal tubular EMT, it can act as an inhibitor of the profibrotic effector cytokine TGF-*β*1. Therefore, they suggested that depending on the cellular microenvironment and/or the specific injury, OSM may indeed have the ability to act as an anti- or profibrotic molecule [11]. Considering the view that OSM might have a dual role in tubulointerstitial fibrogenesis, we treated the MRL/lpr mice with anti-OSM antibody to analyze the mechanism of OSM in TILs in LN mice. In general, different factors have specific STAT response times and answer modes, thus leading to different, even opposite, cellular responses [16, 17]. For example, although IL-27 can simultaneously activate STAT1 and STAT3, the activation of STAT1 rather than that of STAT3 mediates the tumor suppressor role of IL-27 [18, 19]. Moreover, studies by Pan et al. [20] showed that STAT1 mediated OSM-induced suppression of proliferation and migration of lung adenocarcinoma. In contrast, STAT3 showed promoting effects of OSM in lung adenocarcinoma. Combining with our results, we tentatively put forward that the different activation patterns of STAT1 and STAT3 may be interpreted as the dual role of OSM in TILs. It should be noted that this study has examined only the role of anti-OSM antibody in the activation of STAT1 and STAT3. We did not investigate the effect of STAT1 or STAT3 silencing on TILs. Therefore, the dual role of OSM and the coordination between STAT1 and STAT3 in TILs are still waiting to be investigated.

## 5. Conclusion

Through this study, we concluded that OSM is associated with TIL in lupus nephritis, which may be connected with the activation of STAT3 rather than that of STAT1.
